# Chronic IL-21 drives neuroinflammation and promotes lipid accumulation in microglia

**DOI:** 10.1186/s12979-025-00510-2

**Published:** 2025-04-29

**Authors:** Hugo Oyamada, Yinzhi Ying, Sudhanshu Agrawal, Aizhu Liu, Veedamali S. Subramanian, Cleonice Alves de Melo Bento, Anshu Agrawal

**Affiliations:** 1 Division of Basic and Clinical Immunology, Department of Medicine, University of California Irvine, Irvine, CA 92697 USA; 2 Division of Gastroenterology and Hepatology, Department of Medicine, University of California Irvine, Irvine, CA 92697 USA; 3 Department of Microbiology and Parasitology, Federal University of the State of Rio de Janeiro, Rio de Janeiro, Brazil; 4 Department of Microbiology, Immunology and Parasitology, Rio de Janeiro State University, Rio de Janeiro, Brazil

**Keywords:** Neuroinflammation, IL-21, Lipid, Aging, Microglia

## Abstract

Neuroinflammation is a key contributor to the onset and progression of neurodegenerative diseases, driven by factors such as viral infections, autoimmune disorders, and peripheral inflammation. However, the mechanisms linking peripheral inflammation or viral infections to neuroinflammation remain poorly understood, limiting the development of effective therapies. Proinflammatory cytokines are implicated in these processes but their effects on brain cells, including microglia, remain insufficiently characterized. Here, we demonstrate that IL-21, a proinflammatory cytokine elevated in autoimmune disorders, chronic viral infections, and Alzheimer’s disease, activates microglia and promotes lipid accumulation within these cells. Young, healthy mice injected with IL-21 to mimic chronic exposure exhibited increased proinflammatory cytokine levels and microglial activation in the brain. Notably, microglia in these mice displayed enhanced lipid accumulation, accompanied by upregulation of lipid uptake receptors such as CD36 and TREM-2. These findings were corroborated using the human microglial cell line HMC-3, where IL-21 exposure similarly induced lipid accumulation and increased expression of CD36 and ApoE. Mechanistic investigations revealed that IL-21 upregulates HIF-1α, a transcription factor critical for lipid metabolism and lipid droplet formation. Additionally, we observed elevated IL-21 levels in the circulation of elderly individuals compared to younger counterparts, with IL-21 increases associated with CMV seropositivity. Aged mouse brains mirrored the microglial lipid accumulation and activation patterns seen in IL-21-injected mice. In summary, we identify a novel IL-21-driven mechanism involving lipid accumulation in microglia that contributes to neuroinflammation.

**Supplementary Information:**

The online version contains supplementary material available at 10.1186/s12979-025-00510-2.

## Introduction

Neuroinflammation is a critical factor in the onset and progression of neurodegenerative diseases. It is driven by diverse triggers, including viral infections, autoimmune disorders, peripheral inflammation, mental stress, and metabolic disturbances. Despite its recognized importance, the mechanisms linking peripheral inflammation or viral infections to neuroinflammation remain poorly understood. This gap in knowledge hinders the development of effective interventions targeting these pathways.

A key aspect of neuroinflammation involves the role of proinflammatory cytokines, which are elevated during systemic and CNS inflammation. Their impact on central nervous system (CNS) cells, particularly microglia, remains inadequately characterized. Recent evidence points to metabolic changes in microglia, such as lipid accumulation, as a major contributor to their dysfunction [1–4]. These metabolic disruptions are especially relevant in aging and Alzheimer’s disease (AD), where they exacerbate the loss of microglial homeostatic functions, impairing their ability to clear toxic aggregates and cellular debris. Understanding how peripheral inflammation, proinflammatory cytokines, and metabolic dysregulation converge to promote microglial dysfunction is crucial for uncovering the underlying causes of neuroinflammation in neurodegenerative diseases.

Microglia, the resident immune cells of the brain, play a critical role in immune surveillance and maintaining brain homeostasis [5]. They remove cellular debris and pathogens, participate in synaptic pruning, regulate neuronal excitability, and influence astrocyte activation. Given their essential functions, any dysfunction in microglia can lead to brain pathology. Single-cell RNA sequencing studies have revealed that microglia become dysfunctional with aging and in Alzheimer’s disease (AD) [1–3, 6]. In these conditions, microglia adopt an inflammatory state and accumulate lipids, transitioning into disease-associated microglia (DAM). This phenotype is characterized by a loss of their ability to clear damaged cells and toxic protein aggregates along with other changes. However, the underlying mechanisms driving these pathological changes in microglia remain poorly understood.

IL-21 is a proinflammatory cytokine that plays a critical role in the pathogenesis of various diseases. It is markedly elevated in autoimmune disorders such as lupus and multiple sclerosis (MS), where it promotes disease progression by enhancing autoantibody secretion from B plasma cells [7–9]. In chronic viral infections, IL-21 is upregulated to sustain the functionality of CD8 + T cells and prevent their exhaustion, highlighting its dual role in immunity and pathology [10–12].

Our previous studies have shown that IL-21 levels are significantly elevated in the blood of AD and mild cognitive impairment (MCI) patients, where it exacerbates neurodegenerative pathology [13]. Additionally, we have demonstrated that T cells from MS patients with major depressive disorder produce more IL-21 compared to those without depression, suggesting a link between IL-21 and neuroinflammation in comorbid conditions [14]. Other studies further support the involvement of IL-21 in neurological diseases, reporting its increase during acute brain injuries such as stroke [15].

These findings collectively underscore the potential role of IL-21 in driving neuroinflammation and its detrimental effects on brain health. Despite this, the precise mechanisms by which IL-21 contributes to neuroinflammation remain poorly understood. Particularly, its effects on microglial functions, including their inflammatory activation and metabolic dysregulation, are largely unexplored. Here, we investigate the pathways through which IL-21 influences neuroinflammation and microglial dysfunction, shedding light on its role in neurodegenerative and neuroinflammatory disease.

## Results and discussion

### IL-21 enhances neuroinflammation in mice brain

**Fig. 1:**
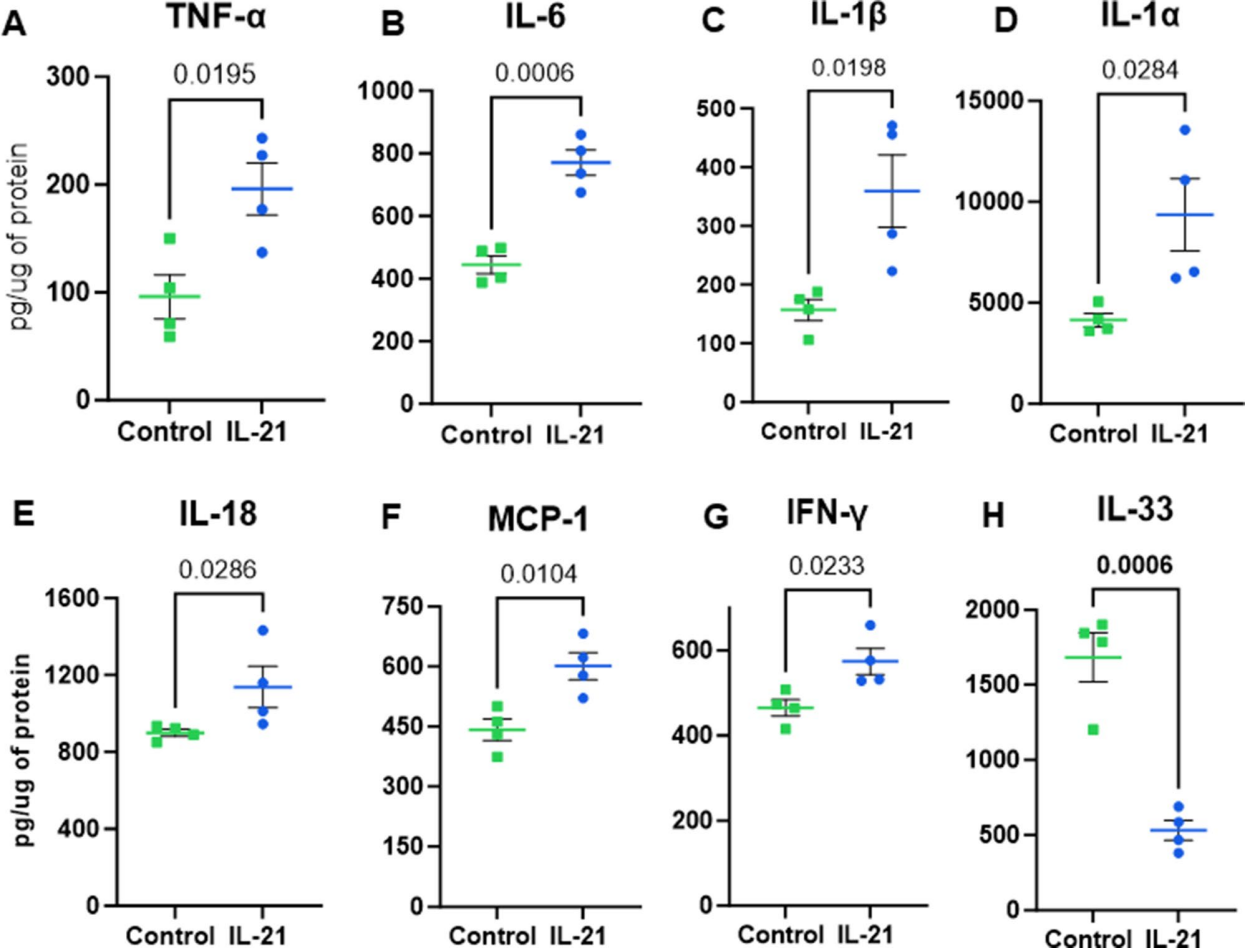
IL-21 enhances neuroinflammation in mice brain. Mice were given 5 injections of IL-21. The level of cytokines and chemokines were determined in the brain using multiplex. Bar graphs depict the pg/µg levels of– (**A**) TNF-α; (**B**) IL-6; (**C**) IL-1β; (**D**) IL-1α; (**E**) IL-18; (**F**) CCL-2; (**G**) IFN-γ.; (**H**) IL-33. Data is Mean +/- S.E

Our previous studies using an Alzheimer’s disease (AD) mouse model demonstrated that IL-21 exacerbates neuroinflammation and AD pathology [13]. However, it remains unclear whether chronic IL-21 exposure induces similar effects in healthy, non-AD mice. This is a critical area of investigation, as elevated IL-21 levels are observed in various autoimmune diseases associated with neuropathology, major depressive disorder, and conditions characterized by chronic inflammation [14, 16–18]. To investigate this, healthy C57BL/6 mice were injected intravenously with recombinant IL-21, and the brain tissue was later assayed for several cytokines using multiplex as described. Our findings reveal that IL-21 injections significantly induced the production of pro-inflammatory markers, including TNF-α, IL-6, IL-1β, IL-1α, IL-18, IFN-γ, and CCL2 (Fig. 1A-G). IL-1β and IL-18, both products of inflammasome activation, are highly inflammatory cytokines. IL-18 plays a critical role in the brain, with activated microglia identified as its primary source [19]. Acting in an autocrine manner, IL-18 stimulates microglia to produce additional pro-inflammatory cytokines, including TNF-α and IL-1β. Moreover, IL-18 induces the expression of Fas ligand (Fas-L) on microglia, oligodendrocytes, and astrocytes, which promotes Fas-mediated neuronal apoptosis during inflammatory states. Activated microglia also produce IL-6, a cytokine that has been found at elevated levels in post-mortem human AD) brains [20]. IL-6 is associated with synapse loss and cognitive deficits. Similarly, TNF-α, another microglial product, contributes to memory impairment [21–23]. In addition to these cytokines, CCL2 is implicated in neuroinflammatory processes, primarily through its role in recruiting glial cells to sites of inflammation [24]. In contrast to these cytokines, the levels of IL-33 displayed significant reduction in IL-21 injected mice (Fig. 1H). IL-33 is constitutively expressed in the brain and spinal cord tissues, where it plays a critical role in maintaining central nervous system (CNS) development and homeostasis. Emerging evidence highlights its function as a mediator of communication between immune cells, endothelial cells, and CNS-resident cells, underscoring its importance in coordinating neuroimmune interactions and supporting CNS integrity. Interestingly, its role in neurological diseases appears context-dependent, as IL-33 can be either protective or detrimental depending on the disease and underlying conditions [25–29].

### IL-21 activates microglia and promotes lipid accumulation within these cells

**Fig. 2:**
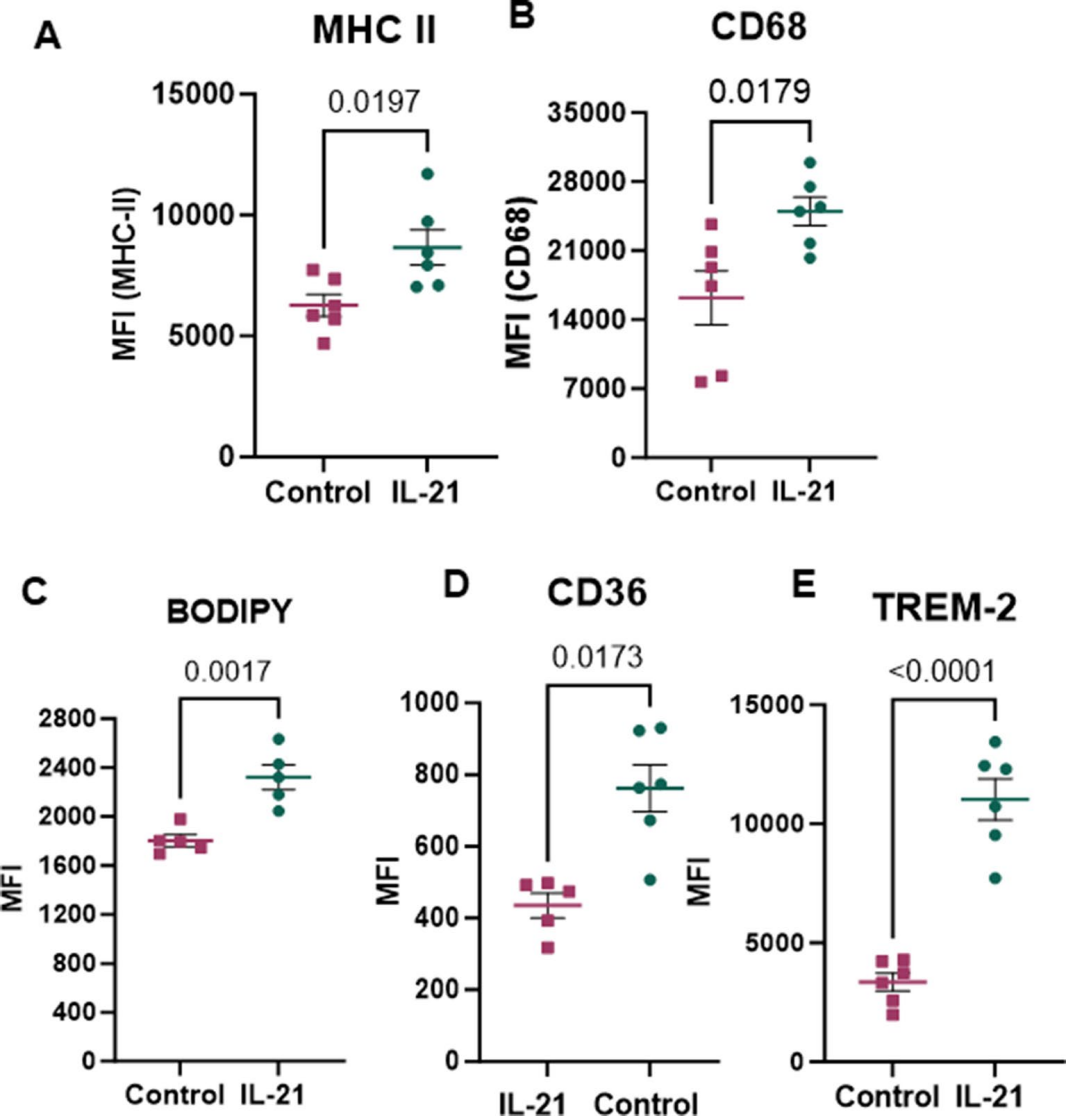
IL-21 activates microglia and promotes lipid accumulation within these cells. Mice were injected with IL-21 twice weekly for total of 5 injections. The activation of microglia and lipid accumulation in microglia was determined by flow cytometry. Dot plots depict the MFI of- (**A**) MHC-II; (**B**) CD68; (**C**) BODIPY; (**D**) CD36; (**E**) TREM-2 on CD45 + CD11b + gated microglia. Data is Mean +/- S.E

IL-21 signals through IL-21R, which is predominantly expressed on microglia, with lower levels detected on astrocytes and neurons in the brain, as shown by the Allen Human Brain Atlas (Supplementary Figure S1). We have also previously reported IL-21R expression on microglia in mice [13]. In this study, we investigated whether IL-21 supplementation could activate microglial cells. To assess this, we stained half-brains from young mice using markers associated with microglial function and activation. Our analysis revealed that IL-21 injections significantly increased the expression of both MHC-II and CD68 on gated microglia (Gating strategy Supplementary Fig. 2) compared to controls (Fig. 2A-B).

Aging and neurodegenerative diseases are known to increase lipid uptake in the brain, contributing to neuroinflammation and neuronal loss [2, 3]. IL-21 has been reported to skew T-cell metabolism towards fatty acid oxidation [30]. To investigate whether IL-21 influences lipid uptake, we compared the uptake of BODIPY which selectively stains neutral lipid droplets [31] between brain microglial cells from IL-21 injected and control mice. We observed a higher lipid content in the microglia of IL-21-injected mice (Fig. 2C). Next, we determined the effect of IL-21 on molecules/receptors involved in lipid uptake. Our findings showed that IL-21 exposure led to a significant increase in the expression of CD36 on gated microglia (Fig. 2D). CD36, a class B scavenger receptor plays a key role in lipid sensing, uptake, and signaling [32]. It facilitates the uptake of lipids like long-chain fatty acids and binding hydrophobic amyloid fibrils in the AD brain [33]. AD pathology in various mouse models correlate strongly with CD36 expression in microglia [32, 34, 35].CD36 also plays a critical role in the pathogenesis of other neurodegenerative diseases such as multiple sclerosis [36].

We also observed increased expression of Triggering receptor expressed on myeloid cells 2 (TREM-2) (Fig. 2E), a transmembrane receptor expressed on microglia in the brain and macrophages in the periphery [37]. TREM-2 regulates inflammatory signaling and microglial metabolism, promoting microglial phagocytosis, activation, survival, and proliferation [38]. It is crucial for normal immune function and cell viability in the brain and is a confirmed genetic risk factor for AD [39]. TREM-2 also plays a vital role in both central and peripheral lipid metabolism. In the CNS, it affects cholesterol and myelin metabolism, binds to phospholipids, and influences their metabolism [40]. TREM-2 promotes the transition of microglia to disease-associated microglia (DAM) through lipid-related pathways, enhancing lipid metabolism in the CNS [40]. The histograms for these molecules are provided in supplementary figure S3.

### IL-21 treated Human microglial cell line displays similar changes as mice brain

**Fig. 3:**
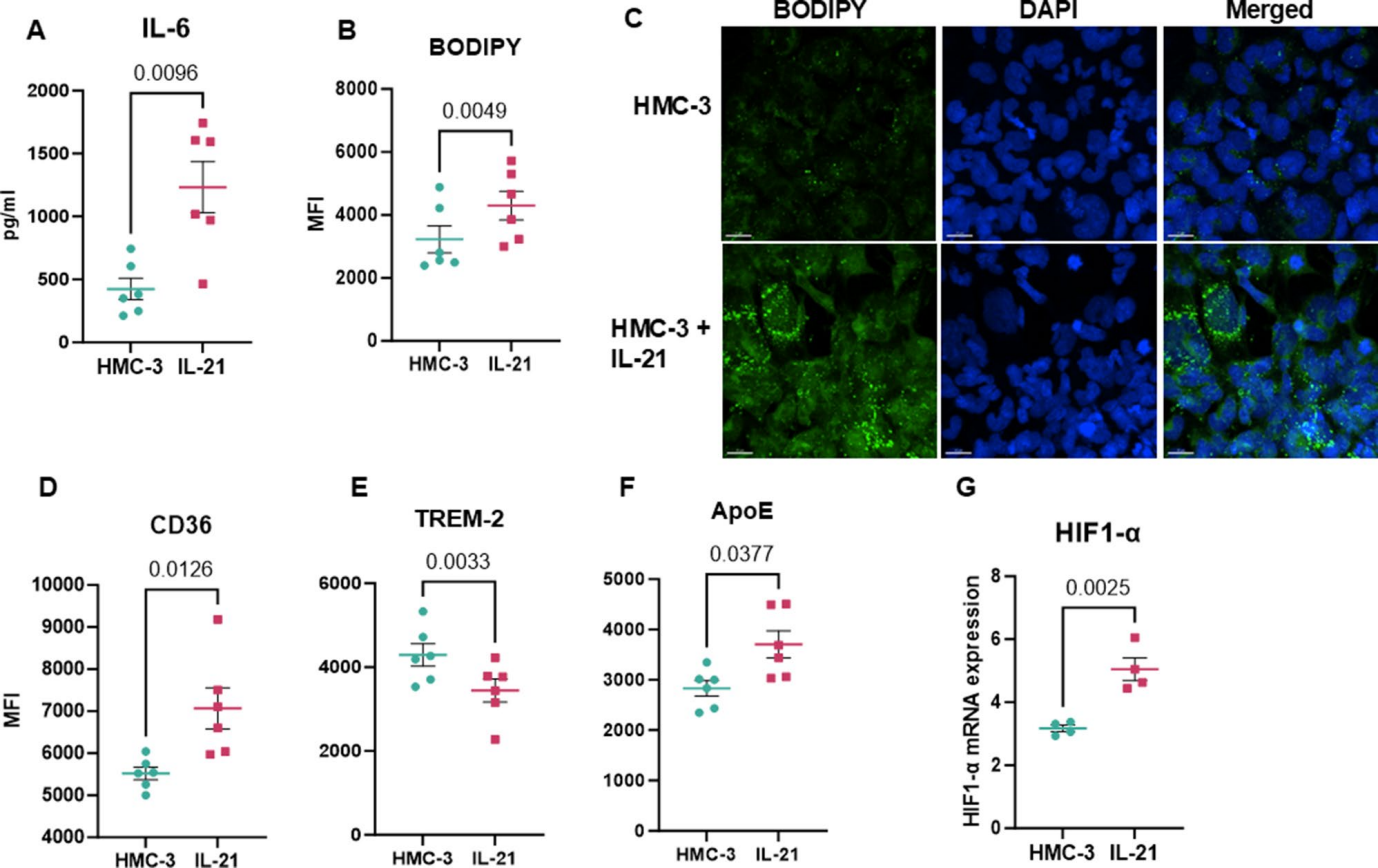
IL-21 treated Human microglial cell line displays similar changes as mice brain. HMC-3 human microglial cell line was exposed to IL-21 for 72 h. (**A**) Supernatant collected was assayed for IL-6. The cells were collected and BODIPY was assessed- (**B**) MFI of BODIPY as determine by flow cytometry; (**C**) Fluorescence microscope imaging of BODIPY (Green) and DAPI (blue); The expression of (**D**) CD36; (**E**) TREM-2; (**F**) ApoE; was determined by flow cytometry. (**G**) Bar graph depicts the mRNA expression of HIF-1α in IL-21 treated cells using q-PCR. Data is Mean +/- S.E

The effects of IL-21 on microglia observed in IL-21-injected mice may result from a direct action of IL-21 on microglia or indirectly through its interaction with other cell types, such as immune cells. Therefore, to determine the direct effect of IL-21 on microglia we utilized the immortalized human microglia cell line, HMC-3 [41]. HMC-3 were exposed to IL-21 for 72 h. After stimulation with IL-21, we found that the cells produced increased levels of IL-6 when compared with unstimulated cells (Fig. 3A) indicating increased inflammation. Next, we determined the effect of IL-21 on lipid accumulation and lipid sensing receptors. We observed significantly increased expression of BODIPY in the IL-21 treated cells both by flow cytometry and fluorescence microscopy (Fig. 3B & C). The expression of CD36 was also significantly upregulated on IL-21 treated microglia (Fig. 3D). Interestingly, in this in vitro model, we found no significant difference in TREM-2 on the cells stimulated with IL-21 (Fig. 3E). However, we observed increased expression of another lipid sensing receptor, the ApoE (Fig. 3F) that interacts with TREM-2 [42]. ApoE mediates cholesterol clearance through binding to various receptors expressed on microglia, such as low-density lipoprotein receptor (LDLR), LDL receptor-related protein 1 (LRP1), and TREM2 [4, 43–46]. Recent scRNA sequencing studies have found that microglia upregulate, the ApoE expression in human and mice brains with AD pathology [47].

Several transcription factors, including PPARγ and HIF-1α, play crucial roles in lipid metabolism and transport in macrophages, with most insights derived from studies on liver diseases and atherosclerosis. We investigated whether IL-21 treatment upregulates these transcription factors and found that IL-21 significantly increased HIF-1α expression (Fig. 3G). Studies have shown that HIF-1 activity is positively associated with lipid accumulation [48–50]. For example, in cardiac myocytes, acute hypoxia redistributed CD36 to the plasma membrane, increasing fatty acid uptake, a finding confirmed in Langendorff-perfused hearts [51]. Hypoxia also enhanced intracellular lipid accumulation by suppressing fatty acid β-oxidation and increasing lipid droplet number and size [52, 53].

### IL-21 levels are increased in aged subjects and microglia in aged mice exhibit alterations reminiscent of those observed in the microglia of mice injected with IL-21

**Fig. 4:**
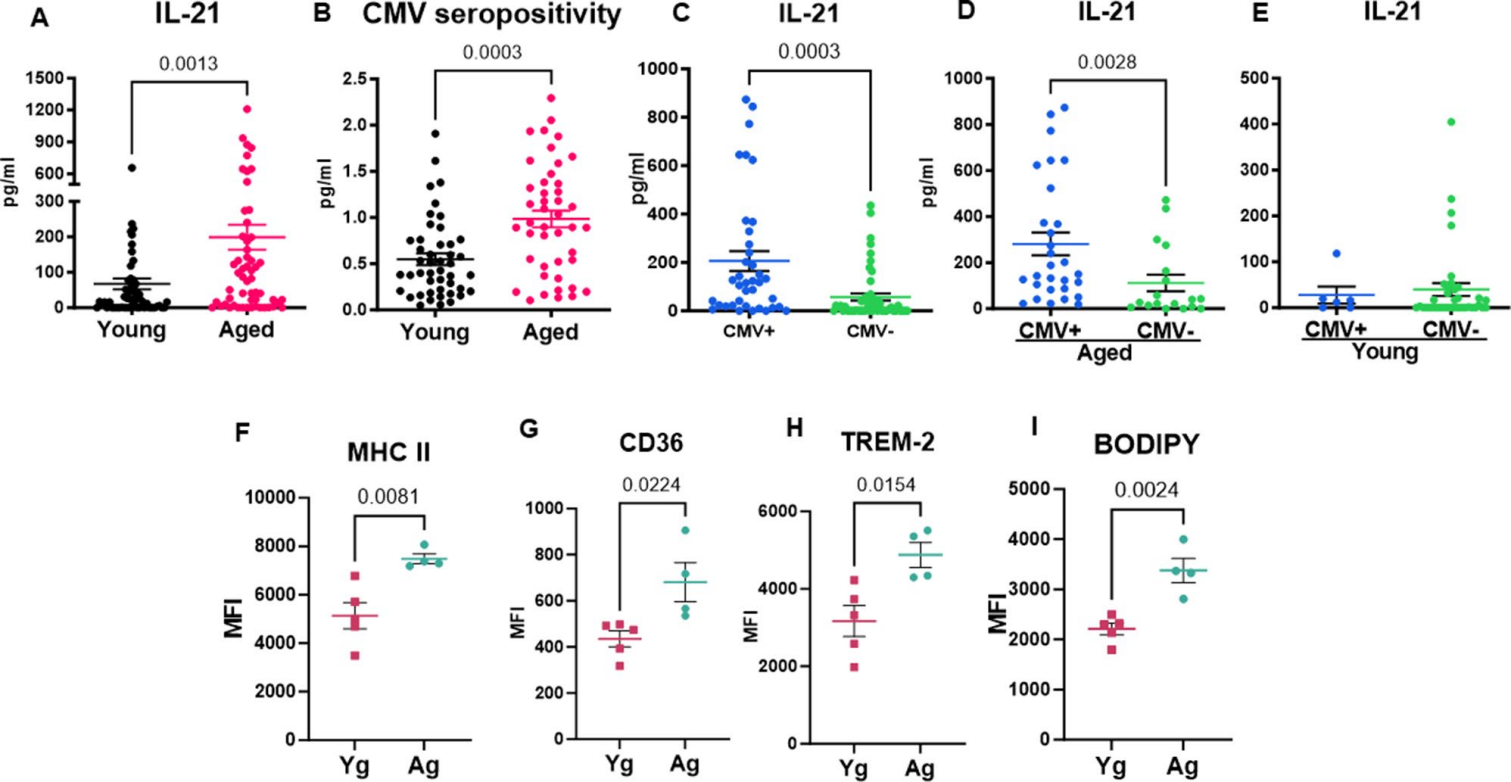
IL-21 levels are increased in aged subjects and microglia in aged mice exhibit alterations reminiscent of those observed in the microglia of mice injected with IL-21. Plasma was collected from aged and young subjects. **A**. Dot plots depict the levels of IL-21 in the plasma of aged and young subjects as determined by ELISA. **B**. CMV seropositivity in the plasma of aged and yound subjects. **C**. IL-21 levels in CMV + and CMV- individuals; **D**. IL-21 levels in CMV + and CMV- aged individuals; (**E**) IL-21 levels in CMV + and CMV- young individuals; Brain cells from aged and young mice were stained with flow cytometry for activation an lipid markers. Graphs depict MFI of- (**F**) MHC-II; **G**.CD36; **H**. TREM-2; **I**. BODIPY

Chronically elevated systemic IL-21 levels are observed in autoimmune diseases and neurological disorders, including AD [7–9, 13, 17]. Systemic IL-21 levels are also increased in major depressive disorders with or without autoimmune diseases [14, 16]. Similarly, chronic IL-21 elevation in circulation is found in individuals with persistent viral infections, where it plays a critical role in preventing CD8 T cell exhaustion both in the periphery and in the brain [10–12]. Given that aging is often accompanied by chronic viral infections, which profoundly impact age-related immunity and disease susceptibility, we investigated IL-21 levels in circulation by comparing plasma samples from aged individuals (> 65 years) and young individuals (21–40 years). As reported by us before [54], a significant increase in IL-21 levels was observed in the elderly as compared to their young counterparts (Fig. 4A). We had previously showed that CMV seropositivity was related with increased IL-21 secretion by CD4 + T cells [54]. Here, we found that CMV seropositivity was indeed increased in aged subjects, when compared with younger individuals (Fig. 4B). Interestingly, when stratified by CMV seropositivity or not, we found that CMV + individuals produced more IL-21 (Fig. 4C). When we dividied the CMV seropositive individuals based on age, IL-21 was increased only in the aged group, with the seropositivity not affecting the cytokine secretion in the younger cohort (Fig. 4D and E).

Since we observed an increase in IL-21 with age, we investigated whether microglia from aged mice (14 months of age) exhibit changes similar to those observed following IL-21 injection. Microglia from aged and young mice were analyzed for the expression of MHC-II, CD36, and TREM-2. Aged microglia showed a significant increase in the expression of all these molecules compared to young microglia (Fig. 4F-H). Additionally, we assessed lipid content using BODIPY staining, which revealed a marked increase in lipid accumulation in aged microglia compared to their younger counterparts (Fig. 4I). Thus, microglia from aged mice displayed similar changes as mice injected with IL-21.

## Conclusion

In summary, these findings reveal a novel mechanism through which IL-21 exacerbates neuropathology. Chronic elevation of IL-21 not only amplifies neuroinflammation but also disrupts microglial function by promoting lipid accumulation, driving them toward dysfunction. Reports indicate that lipid-droplet-accumulating microglia (LDAMs) are cells that accumulate lipid droplets and are associated with a dysfunctional and proinflammatory state in the aging brain [2]. LDAM cells are defective in phagocytosis, produce high levels of reactive oxygen species, and secrete proinflammatory cytokines. The lipid laden microglia in aged mice also display impaired responses in stroke [55]. They have also been observed in human and mouse brains during neurodegenerative diseases. The number of lipid bodies were negatively correlated with cognitive performance, as measured by the mini-mental state exam (MMSE) and positively correlated with Aβ plaque amounts and Tau pathology levels [1]. Recently it was demonstrated that lipid laden microglia when cultured with neurons cause their death [1]. In conclusion, these findings underscore the critical role of IL-21 in driving microglial dysfunction through lipid accumulation, highlighting its potential contribution to neuroinflammation and neurodegenerative processes.

## Materials and methods

### Human subjects

Blood was obtained from aged (65–90 years) and young (20–40 years) subjects. The adult participants (65 years and older) were recruited from the Laguna woods senior center while the young subjects were recruited from UC Irvine. The seniors at Laguna woods live independently. Blood was collected by Immunology and Allergy fellows. The participants were asked to fill out a questionnaire reporting their medical problems which included the list in the Table 1. None of the participant reported any CNS related problems. Exclusion criteria included presence of any diseases affecting the immune system icluding immune disorders or autoimmune disease, infectious diseases, malignancy, diabetes, heart disease, and asthma. In addition, subjects with current use of medications known to affect immune response, including but not limited to immunosuppressants, corticosteroids, biologics, certain antidepressants or antipsychotics with known immunomodulatory effects were also excluded. All elderly subjects were also required to discontinue any anti-oxidants (if they were taking any) at least one week prior to the blood donation. This study was approved by the Institutional Review Board of the University of California, Irvine. Cohort description is provided in Table 1).

**Table 1:**
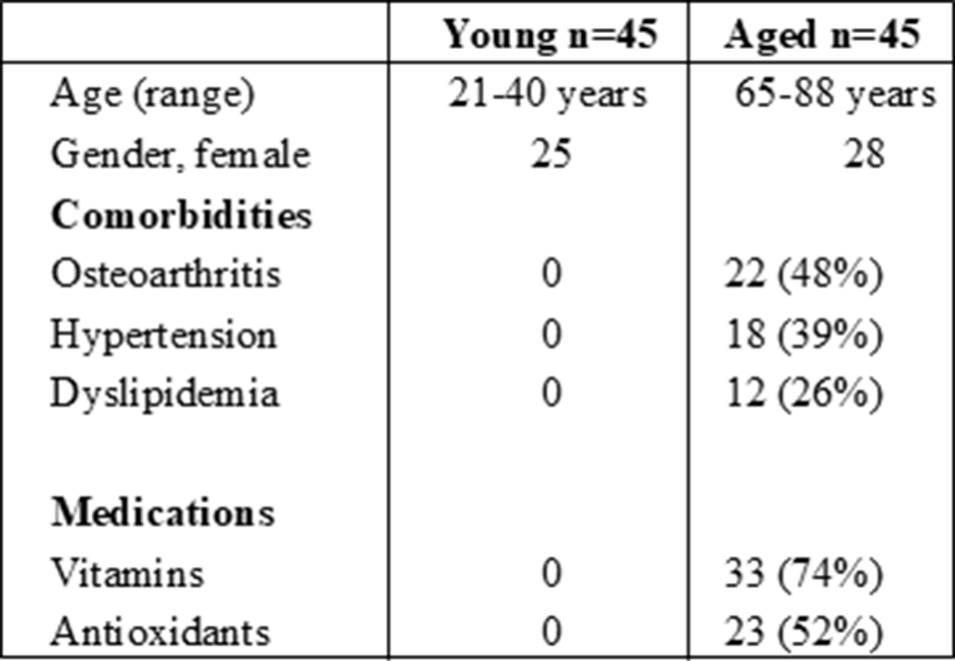
Cohort description

### Plasma collection

Blood was collected in heparin tubes and processed as soon as it was received (less than one hour after blood draw). The blood was centrifuged at 2000 rpm for 15 min and plasma was collected. Collected plasma was aliquoted and kept at -80^o^C until use.

### Mice IL-21 injection experiments

C57BL/6 mice purchased from Jackson Laboratories were used for the study. All animal experimentation procedures were performed in accordance with the guidelines provided by National Institutes of Health (NIH) and approved by the University of California Irvine Institutional Animal Care and Use Committee. Animals were maintained in standard housing conditions (20 °C ± 1 °C; 70% ± 10% humidity; 12 h:12 h light and dark cycle) and provided ad libitum access to standard rodent chow and water. Healthy 3-month-old C57BL/6 mice were injected intravenously with recombinant mouse IL-21 (50 µg/kg) (BioLegend, San Diego, CA), twice weekly for a total of 5 injections as described [13]. Control mice were injected with PBS. 48 h after the last injection, mice were euthanized via intracardiac perfusion using phosphate buffered saline. The brains were collected for analysis. Half of the brain was used for flow cytometry analysis and the other half was flash-frozen in liquid nitrogen for later cytokine analysis.

### Cell lines and culture

HMC-3 cell line was obtained from ATCC and cultivated in Eagles minimum essential media (EMEM) (ATCC, ), supplemented with 10% fetal bovine serum (FBS) and 1% penicillin/streptomycin, at 37 °C in a humidified incubator containing 5% CO_2_. For stimulation, recombinant IL-21 (BioLegend, San Diego, CA) in PBS was added in the cultures, at concentration of 10 ng/ml for 72 h. As a vehicle control PBS was added in the control cells. The cells were subsequently scraped from culture plates, washed, and used for flow cytometry.

### IL-21 and CMV plasma assay

Plasma IL-21 levels from donors were determined by specific ELISA, following manufactures instructions (RnD Systems, Minneapolis, MN). For CMV levels, specific IgG ELISA kits (Cortez Diagnostics Inc, Los Angeles, CA) were used, with the CMV antibody index being determined [54].

### Mouse brain cytokine assay

Previously frozen mice brains were pulverized on dry ice and solubilized in PBS containing a protease inhibitor cocktail. Following centrifugation, supernatant was collected, and cytokine production was determined by Multiplex assay, utilizing a magnetic bead-based customized kit (ThermoFisher Scientific, Waltham, MA) as described [13]. The kit could identify the mediators IL-1β, IFN-γ, IL-17, IL-1α, IL-6, CCL-5, TNF-α, CCL-2, IL-33, and IL-18. The levels of said mediators were then normalized to the amount of protein in the lysate as described [13].

### Flow cytometry

For flow cytometry analysis, half-brains of mice were digested in collagenase and myelin was removed by Percoll density gradient centrifugation. The cells were then collected and stained with BD Horizon™ Fixable Viability Stain 510 for identification of live cells, as per manufacturer’s instructions. Cells were then washed, and surface stained for CD45, CD11b, MHC-II and CD36. After staining, cells were washed and fixated/permeabilizated with BD Cytofix/Cytoperm™ fixation/permeabilization kit (Becton-Dickenson, San Jose, CA), following the provided instructions. After washing, cells were intracellular stained for CD68, ApoE and TREM-2 markers. Acquisition was done on BD FACS Celesta (Becton-Dickenson, San Jose, CA). Forward and side scatters were utilized to gate and eliminate cellular debris. Analysis was performed using FlowJo™ 10.10 software (BD Life Sciences, Ashland, OR).

For BODIPY staining brain cells prepared as above were incubated with BODIPY (boron-dipyrromethene) 493/503 dye for 15 min at 37 °C. Subsequently they were washed and stained for surface markers. Cells were then washed, acquired on a flow cytometer, and analyzed for lipid content in gated microglia. For HMC-3 cells were first collected and then stained as above.

### Fluorescence microscopy

The BODIPY stained HMC-3 were scanned using a confocal microscope (Nikon Eclipse Ti C2) equipped with a 40× PlanApo oil-immersion lens (1.3 NA, Nikon) and an NIS-Elements AR interface (v4.30, Nikon). 20–30 z stacks (1024-bit depth) at 0.5 μm from three different fields (318 × 318 × 24 μm) were imaged in each section in the areas of interest. The digitized z stacks were deconvoluted using the AutoQuant software (version X3.0.4, Media Cybernetics, Rockville, MD).

### Statistical analysis

For statistical analysis, GraphPad Prism 10.0 software was utilized. Paired and unpaired Student’s *t* test was used, when applicable. Values of *p* < 0.05 were considered significant. All tests were two tailed with 95% confidence interval.

## Electronic supplementary material

Below is the link to the electronic supplementary material.

Supplementary Material 1

## Data Availability

No datasets were generated or analysed during the current study.
