# Supplementary material for: Chronic IL-21 drives neuroinflammation and promotes lipid accumulation in microglia
Source: Immun Ageing. 2025 Apr 29;22:15. doi: 10.1186/s12979-025-00510-2 (PMC12039274; doi:10.1186/s12979-025-00510-2)
Supplement: Supplementary file 1 — Supplementary Material 1 [file 12979_2025_510_MOESM1_ESM.pdf]

Supplementary Figure S1

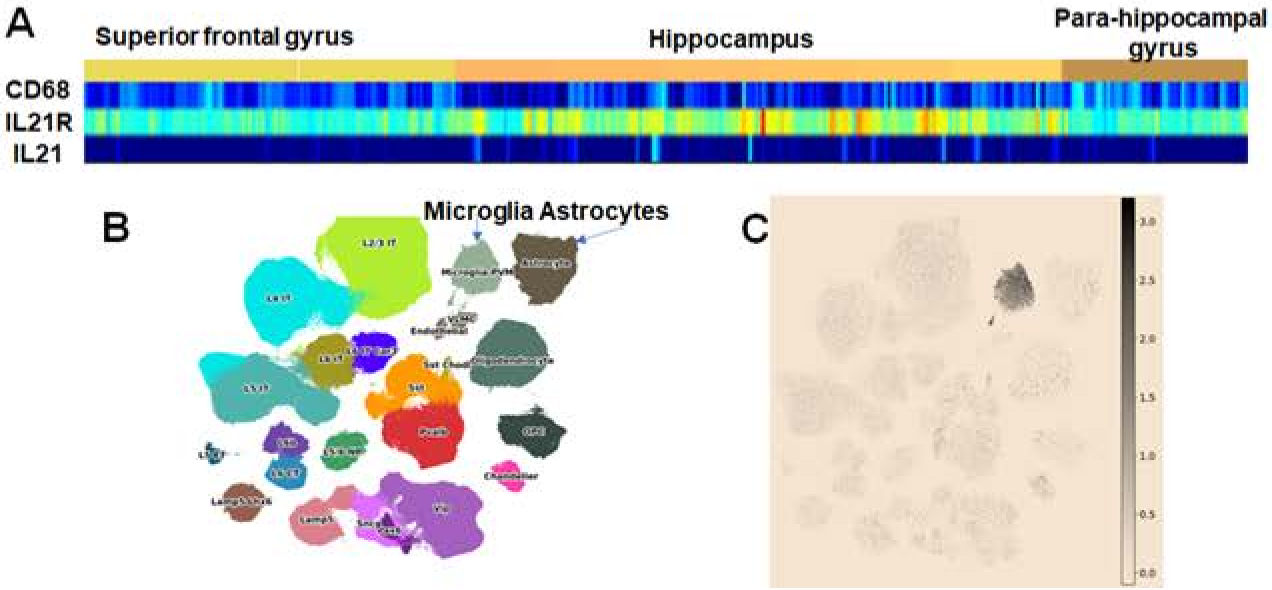

Supplementary Figure S1: IL-21R expression is highest in the microglia of human brain. A. Microarray data from human brain showing expression of CD68, IL-21R and IL-21 in different brain regions. **B.** Single nuclei sequencing data showing cells by color. **C.** IL21R expression showing significantly higher expression in microglia but detectable levels of expression in other cell types in the CNS. **Data for the figure is from ALLEN brain atlas.**

Supplementary Figure S2

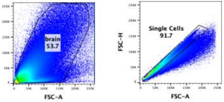

Gating strategy for microglia

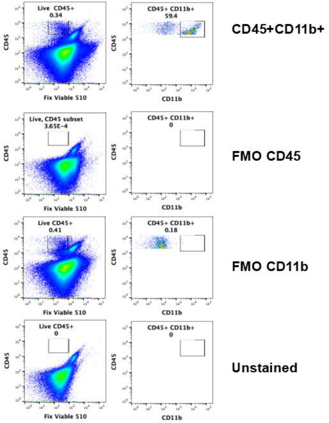

Supplementary Figure S2

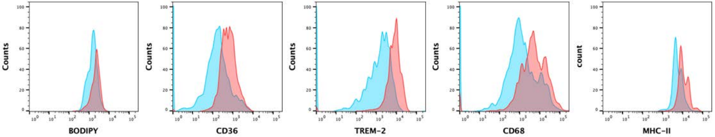

Gated microglia (figure S2) from IL-21 injected and control mice were analyzed for the expression of BODIPY, CD36, TREM-2, CD68 and MHC-II by flow cytometry. Histograms depict the MFI of the same. Red- IL-2; Blue- Control. Representative of 6 experiments. Bar graph is presented in Figure 2.
